# Alport Syndrome With a Rare Collagen Type IV Alpha-4 (COL4A4) Gene Mutation: A Case Report

**DOI:** 10.7759/cureus.69187

**Published:** 2024-09-11

**Authors:** Akshaya Rana, Surekha Tayade

**Affiliations:** 1 Department of Obstetrics and Gynaecology, Jawaharlal Nehru Medical College, Datta Meghe Institute of Higher Education and Research, Wardha, IND

**Keywords:** alport syndrome, autoimmune hypothyroidism, col4a4 gene, hematuria, keratoconus

## Abstract

Alport syndrome (AS) is a rare, progressive hereditary kidney disease characterized by sensorineural hearing loss and visual abnormalities. It is caused by a mutation in the collagen type IV alpha-4 (COL4A4) gene, which produces type IV collagen, and often manifests in individuals with hematuria, proteinuria, edema, and hypertension. Here, we present a case of AS in a 15-year-old boy with a COL4A4 gene mutation, with renal and extrarenal findings. The patient presented with subnephrotic proteinuria and microscopic hematuria, autoimmune hypothyroidism, and keratoconus. Light microscopy examination of renal biopsy revealed three globally sclerosed renal cortical parenchyma areas with periglomerular fibrosis, and electron microscopy showed variable thickness of glomerular basement membrane with festooned appearance, as well as splitting of lamina densa giving basket weave and criss-cross pattern.

## Introduction

According to certain theories, the modern era of Alport syndrome (AS) began in the 1970s with observations of distinctive ultrastructural anomalies in the glomerular basement membranes (GBMs) of affected individuals [1-3]. These ground-breaking findings sparked a series of inquiries that resulted in the discovery of collagen IV (COL4) as the protein responsible for AS. Collagen type IV alpha 3 (COL4A3), collagen type IV alpha 4 (COL4A4), and collagen type IV alpha 5 (COL4A5) genes cloning and sequencing, and the identification of variations in these genes in AS families [4-8]. The alpha 3, alpha 4, and alpha 5 chains of COL4 are encoded by the genes COL4A3, COL4A4, and COL4A5, respectively.

The main element of the kidney's GBM is COL4. COL4 alpha chains are mainly found in the kidneys, eyes, and cochlea. The splitting of the GBM or more serious pathological lesions affecting the kidney is caused by pathogenic mutations involving this gene [9]. These genes have three different inheritance patterns and more than 1,700 mutations. Three inheritance types are X-linked Alport syndrome (XLAS), autosomal recessive Alport syndrome (ARAS), and autosomal dominant Alport syndrome (ADAS).

This condition is characterized by progressive nephritis with GBM ultrastructural alterations, hematuria, and proteinuria, ultimately leading to kidney failure. Usually, extra-renal manifestations such as sensorineural hearing loss and ophthalmologic illnesses like lenticonus or retinal flecks are present [10]. It is crucial to diagnose AS as soon as possible, as its consequences typically appear in childhood or early adulthood, and renal disease can lead to death in early adulthood if treatment is not started on time [11].

Technology has progressed to the point where a variety of different research methods, in addition to electron microscopy, have increased the number of clinical and research applications to identify changes in AS [12]. These methods include genetic testing and immunohistochemistry (IHC) investigation of the expression of the basement membrane COL4 by skin or renal biopsy [13].

The first and most important factor in treating AS is managing and delaying the progression of renal damage. The doctor will prescribe appropriate treatment like angiotensin-converting enzyme (ACE) inhibitors, beta-blockers, calcium channel blockers, and a low-sodium and low-protein diet. When kidney disease reaches end-stage renal disease, dialysis or kidney transplantation are the only treatment options available.

## Case presentation

**Figure 1:**
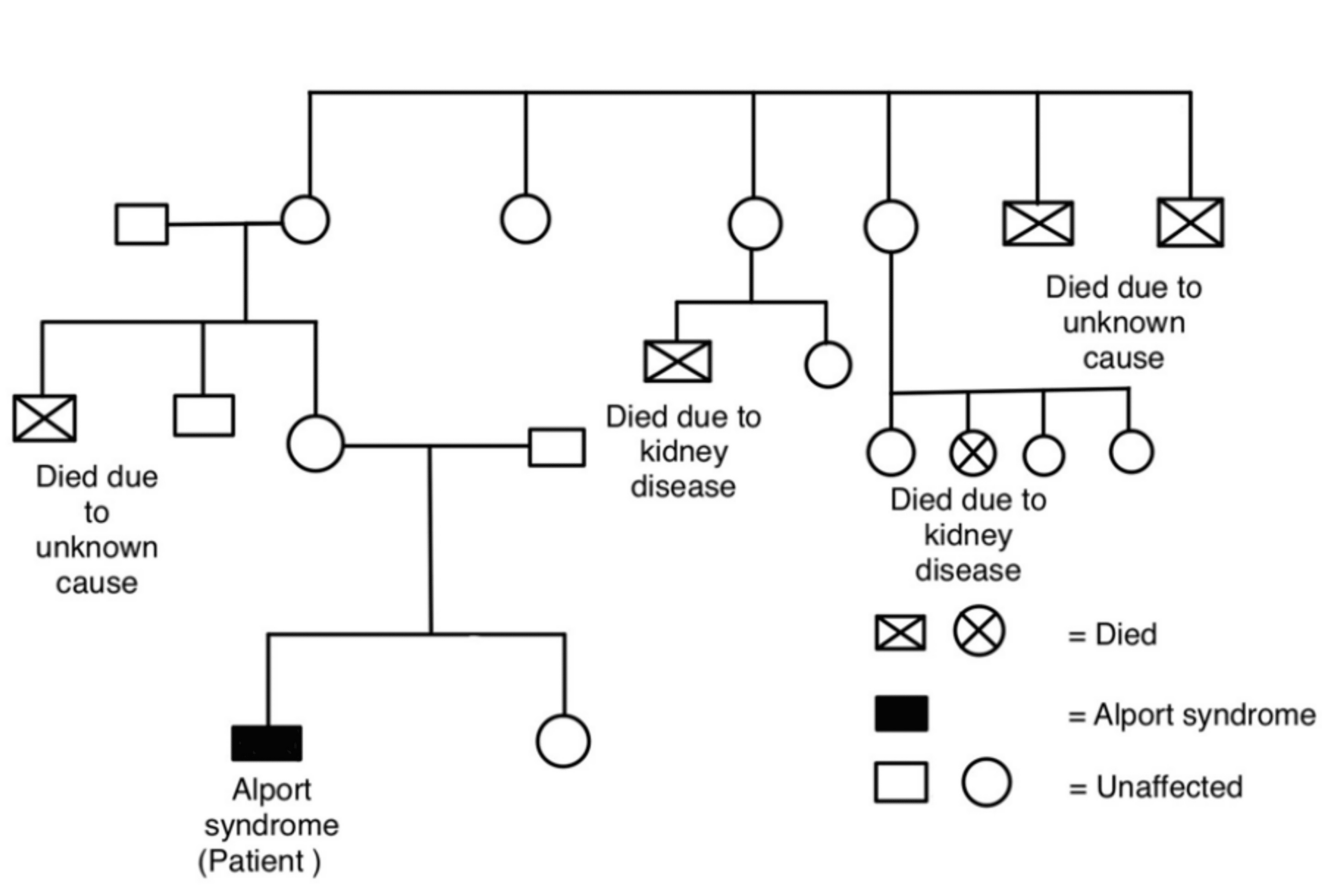
Family pedigree chart

The patient is a 15-year-old boy who was found to have subnephrotic proteinuria with microscopic hematuria, autoimmune thyroiditis, and keratoconus at the age of 11 years. He had a history of sickle cell trait, AS pattern, and he was operated on for phimosis when he was seven years old. At the age of eight years, he was diagnosed with bilateral hydrocele and was referred to a pediatric surgeon. When he was 12 years old, he developed an acute kidney injury due to an acute viral infection, which was cured. At 14 years, on clinical examination, it was found that the patient had micropenis (length = 44 mm) and cushingoid facies and skin lesions over his face; he was moderately anemic (9 gm/dL). The patient had 24 hours of urine protein was 567 mg/24 hrs, which increased gradually over a few months to 879 mg/24 hrs; creatinine levels were 1.83 mg/dL. His urinalysis demonstrated reddish urine with hazy transparency, with pus cells 50-100/hpf (high power field), 3+ proteinuria, protein/creatinine ratio 6.25, and 3+ urobilinogen. Ocular examination revealed corneal ectasia and keratoconus in both eyes. Based on the subnephrotic range of proteinuria and subsequent discussion with the patient's mother, it was discovered that the patient's maternal second uncle and aunt had passed away due to kidney disease (Figure 1).

**Figure 2:**
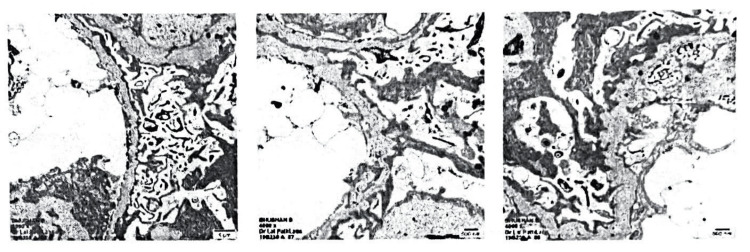
Renal biopsy showing "basket weave" like appearance and "criss-cross" patterns in glomerular basement membrane

In March 2019, ultrasonography, renal biopsy, and genetic testing were done (Figure 2). On ultrasonography, bilateral small kidneys (7.1 x 3.4 cm right; 7.2 x 3.3 cm left) with raised cortical echotexture, loss of corticomedullary differentiation, and grade 3 renal parenchymal disease with splenomegaly were noted. Renal biopsy showed prominent alterations in the arrangement of collagen in GBMs, including the splitting of GBM, "basket weave"-like appearance, and "criss-cross" patterns, and widespread significant effacement of foot processes of visceral epithelial cells (about 60-70%). Variability of GBM thickness with thin and thick areas (maximum and minimum GBM thickness being 121.1 and 918.3 nm, respectively) confirmed the diagnosis of AS (Figure 2). According to genetic testing, the patient had a heterozygous intronic mutation in the COL4A4 gene, where thiamine was substituted for guanine at nucleotide 871 (c871-72G > T and c871-64A > T). This mutation is expected to modify the splicing sites and final protein product. In October 2022, the patient presented to the hospital complaining of generalized weakness, gastritis, and edema around the eyes. His clinical examination revealed blood pressure 130/80 mmHg, blood urea 156 mg/dL, creatinine 9.6 mg/dL, potassium 5.2 meq/L, and urinalysis showed 2+ proteinuria, pus cells 1-2/hpf. The treatment was started when the patient was nine years old, and rabeprazole and telmisartan were given initially. The patient's condition deteriorated over the years, for which the patient is currently on dialysis and given supportive care treatment such as Thyrox 50 mcg once daily (thyroxine), Amlokind 5 mg once daily (antihypertensive), Dilzem 30 mg twice daily (calcium channel blocker), C2D3 (calcium and vitamin supplement), Sobisis 500 mg (antacid).

## Discussion

AS is an uncommon condition that leads to progressive nephritis. In 85% of instances, an X chromosomal aberration linked to the COL4A5 gene has been found. The youngster in this case was diagnosed with AS based on the findings of a kidney biopsy and genetic testing, due to recurrent observations of severe hematuria and proteinuria. Improvements in renal biopsy and genetic testing have made kidney disease diagnosis and treatment more accessible [14,15]. In 15% of cases of AS, ARAS is caused by mutations in the COL4A3 and COL4A4 genes [16,17]. COL4A3 and COL4A4 gene mutations are present in 5% of people with ADAS. AS affects the integrity of the basement membrane of the eye, cochlea, and glomerulus due to a disruption in the COL4A5 gene. In the AS kidney, longitudinal GBM splitting develops later, and the basement membrane weakens earlier under electron microscopy. Recurrent bouts of gross hematuria are one of the clinical symptoms, especially in young children.

Typically, asymptomatic microhematuria occurs before other symptoms. In the early stages of the condition, plasma creatinine levels and blood pressure remain normal; however, later on, hypertension, azotemia, and proteinuria may develop. In the later stages, patients may present with end-stage renal disease. Eye and ear involvement are the most common extrarenal features. Hearing conversational speech becomes increasingly difficult over time due to sensorineural hearing loss, which initially affects high-frequency sounds. Eye changes include anterior lenticonus, white or yellow flecking of the perimacular portion of the retina, and corneal lesions such as recurrent corneal erosions, posterior polymorphous dystrophy, and, very rarely, keratoconus [9]. Diagnosis of AS is typically supported by a thorough family history of renal illness, as done in this case. However, in 15% of cases, there is no family history, and a kidney biopsy is used for diagnosis. A renal biopsy examining COL4 expression in the kidney can confirm or rule out AS. Supportive care includes medications such as ACE inhibitors and calcium channel blockers to reduce proteinuria and control hypertension. Patients with end-stage renal disease also receive dialysis. Research into AS gene therapy is ongoing, with current studies involving the administration of the human alpha-5 (IV) chain of GBM in a canine model of XLAS [18]. In this case, the boy also presented with autoimmune thyroiditis. To date, there is no evidence or research linking autoimmune thyroiditis with AS.

## Conclusions

AS is a rare disorder, which makes it a compelling case to investigate different approaches to therapy. It presents with distinct clinical symptoms, including renal failure, visual involvement, and hearing loss. Here, we report a rare case of XLAS in a 15-year-old adolescent boy with an unusual extrarenal clinical manifestation of keratoconus, along with autoimmune hypothyroidism and sickle cell trait. Symptomatic relief can be achieved with a multidisciplinary approach and supportive therapy, as there is no cure for this illness. The patient received maintenance dialysis and supportive care and treatment. Early diagnosis of the disease allows for appropriate genetic counseling for both the patient and other family members.
